# Exploring the relationship between social media use and loneliness in adolescents and young adults: a systematic review and meta-analysis

**DOI:** 10.1007/s00127-026-03175-4

**Published:** 2026-07-21

**Authors:** Hsin-Pei Feng, Yin-Ju Lien, Sih-Yu Cian, Wei-Ya Wang, Ying-Ting Li, Chao-Hui Chen, Yi-Yun Wu

**Affiliations:** 1 Department of Health Promotion and Health Education, National Taiwan Normal University, 162, Section 1, Heping E. Rd, Taipei, 106 Taiwan; 2 College of Nursing, National Defense Medical University, Taipei, Taiwan

**Keywords:** Social media use, Loneliness, Adolescents, Meta-analysis

## Abstract

**Purpose:**

As social media becomes increasingly integrated into adolescents’ daily lives, concerns have grown regarding its potential association with loneliness, a prevalent challenge during this developmental period. This meta-analysis examines the relationship between social media use and loneliness, with particular attention to different patterns of engagement.

**Methods:**

A systematic search of six databases was conducted for studies published between January 2000 to May 2025. Fifty-nine eligible studies were identified, comprising 67 independent samples with a total of 81,344 participants (59.5% female; mean age = 17.6 years, range = 12.5–24.9). Random-effects models were used to compute pooled effect sizes. Meta-regression and subgroup analyses examined potential moderators, including geographic region, gender, and publication year.

**Results:**

Substantial heterogeneity was observed across studies (*I*^*2*^ = 87.86%–97.01%). Meta-regression analyses showed no significant moderating effects of geographic region, gender, or participants’ year. However, problematic social media use showed a moderate and consistent association with greater loneliness (*r* = 0.29, 95% CI [0.22–0.35], *p* < 0.001), whereas time spent on social media demonstrated a smaller but significant correlation (*r* = 0.07, 95% CI [0.01–0.12], *p* = 0.014). No significant differences were found between active and passive use in relation to loneliness.

**Conclusions:**

These findings underscore the importance of distinguishing usage patterns when examining the psychological effects of social media and indicate the need for additional rigorous investigation to clarify the mechanisms underlying its relationship with loneliness in adolescence.

## Introduction

Social media, grounded in Web 2.0 technologies and principles, enables users to create and disseminate content, thereby reshaping contemporary communication dynamics [1]. Evidence indicates that online interaction has become the predominant form of social engagement among adolescents. More than 95% of adolescents report daily use of platforms such as Snapchat, Instagram, YouTube, TikTok, WeChat, and Twitter [2, 3]. By 2022, YouTube had become the most widely used platform, followed by TikTok, Instagram, and Snapchat [4]. These evolving platform preferences highlight the increasingly central role of social media in adolescents’ daily lives and underscore its growing influence on social development.

Loneliness, defined by Perlman and Peplau (1981) [5] as the distress experienced when one’s social relationships are perceived as inadequate in quantity or quality, represents a pressing developmental challenge. Adolescents are particularly vulnerable to loneliness as they navigate expanding peer networks and explore their personal identities [6]. The simultaneous drive for autonomy and the desire for connectedness can further intensify feelings of isolation [7]. The consequences of adolescent loneliness are wide-ranging, including reduced academic achievement [8], increased risk of school dropout [9], and heightened susceptibility to depression and suicidal behaviors [10, 11]. Collectively, these findings highlight the urgent need to address loneliness during adolescence as both a public health and societal priority.

Adolescents experiencing heightened levels of loneliness often adopt passive approaches to social interactions, which may hinder their ability to establish and sustain positive relationships [12]. Social media provides a potential avenue to reshape these interaction patterns. However, evidence suggests that its influence is ambivalent: some studies report that social media use exacerbates loneliness [10, 13, 14], whereas others indicate that it alleviates feelings of isolation [3, 15–17]. Thus, the psychological impact of social media cannot be regarded as inherently detrimental or beneficial; rather, it depends largely on how and in what contexts these platforms are used [12, 18].

Social media use can be conceptualized in multiple ways, one of the most common being time spent, which typically refers to the number of hours per day or week adolescents engage with these platforms [19, 20]. Findings regarding this dimension remain inconsistent. Some studies have documented a positive association, suggesting that greater time online is linked to higher levels of loneliness [21–23]. In contrast, other research has shown a negative association, indicating that increased time on social media can reduce feelings of loneliness [12, 24–26]. These conflicting findings highlight the need for a more systematic evaluation of the relationship between time spent on social media and adolescent loneliness.

The concept of problematic social media use refers to individuals who experience addiction-like symptoms stemming from their engagement with social networking platforms [27]. It is considered a non–substance-related behavioral disorder characterized by preoccupation, compulsive use, and the persistence of such behaviors despite negative consequences [28]. A screen-dominant lifestyle can therefore be costly, and substantial evidence links problematic social media use with increased loneliness [25, 29–31]. Longitudinal studies further suggest that problematic use can prospectively predict heightened loneliness [32, 33].

Nevertheless, alternative perspectives complicate this view. The stimulation hypothesis proposes that problematic use may serve as a form of negative reinforcement by temporarily alleviating loneliness through strengthening existing relationships or facilitating the formation of new ones [34]. More recently, Nowland et al. (2018) [35] argued for a bidirectional and dynamic relationship between loneliness and problematic social media use. Specifically, when engagement functions as an escape from offline social interactions, loneliness tends to intensify. Conversely, when it promotes meaningful social connections, loneliness may be reduced.

In addition to problematic use, researchers have distinguished between active and passive social media use. Active use involves generating content and engaging in direct communication, such as posting photos, sharing updates, or commenting on others’ posts [36]. This form of use is generally associated with lower loneliness, largely because it fosters more frequent and meaningful interactions with close friends [37]. By contrast, passive use—characterized by content consumption without direct interaction, such as scrolling through feeds or viewing others’ posts [38]—is often linked to greater loneliness [22, 23, 39].

However, research suggests that passive social media use may also provide indirect benefits. It can facilitate access to information and resources, particularly for individuals who may lack such support in offline contexts, thereby potentially alleviating loneliness [40, 41]. Fish et al. (2020) [42] further emphasized that for certain groups, such as individuals with special needs within the lesbian, gay, bisexual, transgender, and queer/questioning (LGBTQ) community, passive use offers opportunities to observe and learn from the experiences of others. This vicarious engagement can foster a sense of belonging and support, ultimately reducing feelings of loneliness. Despite these potential benefits, findings on both active and passive use remain inconsistent across studies.

Meta-analytic evidence underscores this complexity. Song et al. (2014) [43] investigated the relationship between Facebook use—including compulsive engagement and time spent on the platform—and psychosocial traits such as anxiety, shyness, loneliness, and extraversion. Their analysis, which included eight studies with 18 effect sizes, revealed a small but significant positive association (*r* = 0.13, *p* < 0.05) between increased Facebook use and higher loneliness. However, the scope was limited, as it focused exclusively on Facebook and did not specifically examine adolescents. Similarly, Liu and Baumeister (2016) [44] conducted a meta-analysis of 23 samples exploring the links between SNS use, self-esteem, narcissism, and loneliness, operationalized as time spent on SNS and Facebook addiction. They reported a weighted mean correlation of 0.17 (*p* < 0.05), with stronger associations observed in studies involving fewer females and participants from non-Western, collectivist cultures. Nonetheless, this study also had limitations: it did not differentiate between types of social media use (e.g., active vs. passive) and was not tailored to adolescent populations.

Considering the diverse effects and directions of the relationship between different patterns of social media use and adolescent loneliness reported across studies, examining potential moderators is essential. This study therefore investigates whether participant region, gender, and age influence these associations. Prior research suggests that regional variations may contribute to inconsistent findings. For instance, Kim et al. (2011) [13] observed that Korean students primarily use social media for social support, whereas American students tend to use it for entertainment. Similarly, prevalence rates of problematic Facebook use (PFU) vary substantially across regions: Lopez-Fernandez et al. (2023) [45] reported rates between 0.8% and 5.8% in Western countries, while Luo et al. (2021) [46] found that approximately 3.5% of Chinese adolescents experienced social media addiction. These discrepancies underscore notable global differences in problematic use, further supported by Zhou et al. (2023) [47].

Gender also plays a significant role in shaping the relationship between social media engagement and loneliness. Coyne et al. (2020) [48] highlighted gender-specific differences in social media usage patterns, which may influence loneliness outcomes. Vahedi (2021) [49], in a meta-analysis, found that women are more likely to experience loneliness when engaging in passive social media use, whereas men tend to benefit from active use through greater social support. Moreover, Liu et al. (2022) [50] demonstrated that time spent on social media is associated with an increased risk of depression, with stronger effects observed in adolescent girls (OR = 1.72, 95% CI [1.41–2.09]) compared to boys (OR = 1.20, 95% CI [1.05–1.37]). These findings collectively suggest that gender may moderate the psychosocial consequences of social media use.

Despite growing interest, current meta-analyses on social media use and loneliness typically examine broad populations, often failing to focus specifically on adolescents [43]. When adolescents are considered, analyses usually address only a single dimension of social media interaction, such as time spent or problematic use [51, 52]. This narrow focus limits understanding of how distinct patterns of engagement, including time spent, problematic use, active use, and passive use, differentially affect adolescent loneliness.

To address these gaps, the present meta-analysis systematically evaluates multiple forms of social media use in relation to loneliness, with an exclusive focus on adolescents, a population particularly vulnerable to social disconnection. By incorporating potential moderators such as region, gender, and age, this study seeks to clarify inconsistencies in the existing literature and provide a more comprehensive understanding of the magnitude and direction of these associations.

## Method

### Literature search, screening, and study selection

We systematically searched electronic databases such as APA PsycArticles, EBSCOhost, ERIC, MEDLINE, PubMed, and Airiti Library for studies published from January 1, 2000, to May 31, 2025. This focus on recent research ensures the incorporation of refined methodologies and contemporary data, reflecting the latest developments in academic and clinical knowledge.

The search strategy incorporated keywords across all fields. For social media components, keywords included “social media,” “social network,” “social network platform,” and “social networking site.” Keywords for social media usage included “frequency,” “duration,” “screen time,” “time spent,” “investment,” “intensity,” “activity,” “experience,” “problematic use,” “addiction,” and “appearance-focused use.” Terms for specific participant groups included “adolescents,” “teen,” and “youth,” “young adult.” For loneliness keywords included “loneliness,” “lonely,” “lonesome,” and “lonel*.”

To identify empirical studies measuring two or more variables of interest, we used Boolean operators (AND, OR, NOT) to refine our search and filter out irrelevant terms. We further broadened our scope by manually examining bibliographies and conducting backward searches. Additionally, we consulted analytical literature referenced in relevant systematic reviews and meta-analyses, focusing on studies published from January 1, 2020, to May 31, 2025, to capture the latest research.

**Fig. 1:**
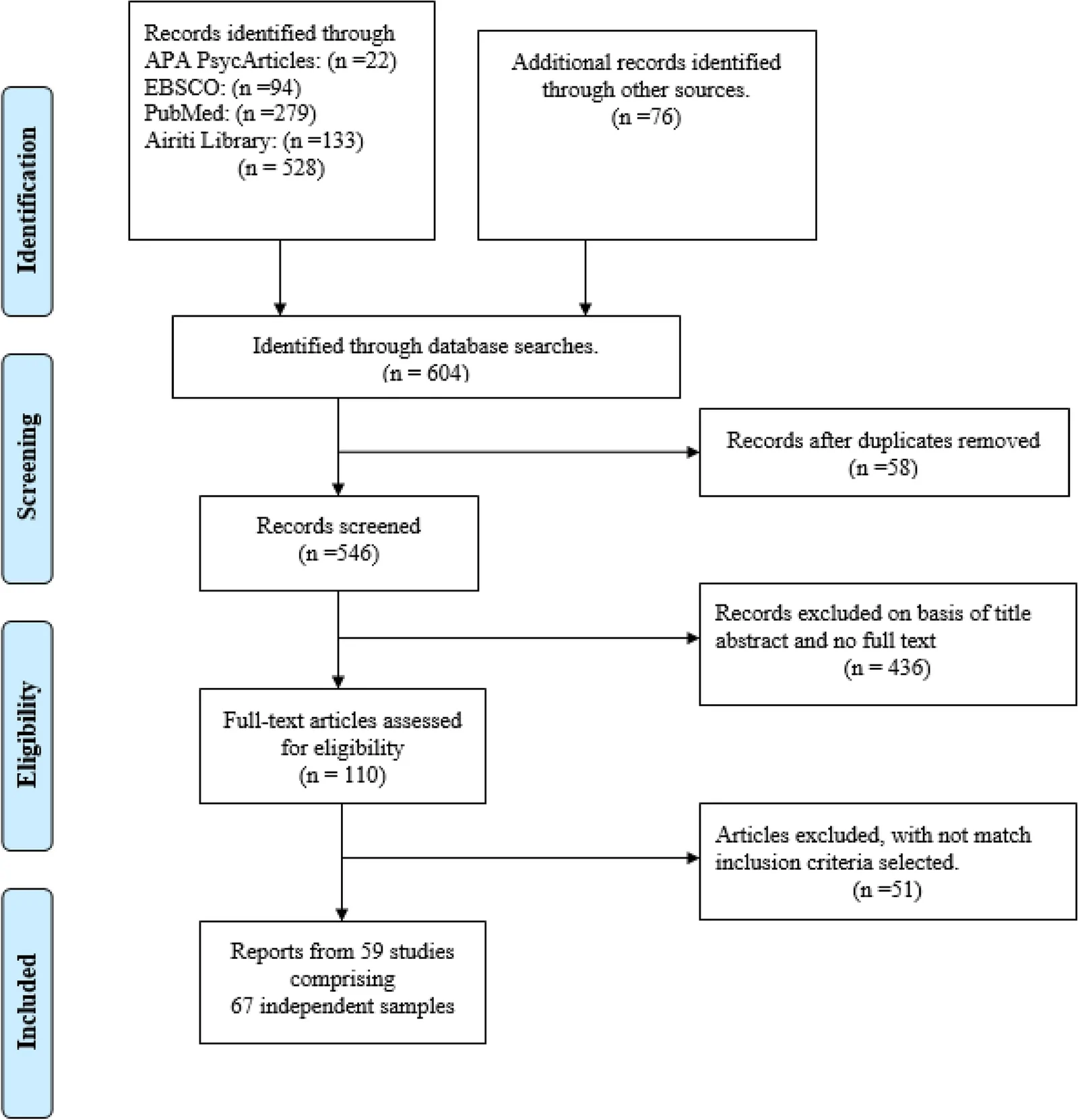
Flow chart of the search process and studies included

Titles and abstracts were screened using predefined criteria, targeting only full-text, peer-reviewed studies in English and Chinese, involving individuals aged 10 to 25. Exclusions applied to review articles, case reports, qualitative studies, unrelated topics, duplicates, and studies centered on interventions, training, or treatment outcomes. Eligibility required studies to present at least one Pearson’s correlation coefficient (*r*) or an equivalent statistical measure (e.g., odds ratio, β coefficient,* t* or *F* value, chi-square value), assessing the relationship between social media use and loneliness, along with specified sample sizes. For data sets utilized in multiple studies, only the most recent publication was considered. Two authors independently conducted the title and abstract screening. Disagreements were initially resolved through discussion; persistent issues were referred to a third author for final resolution. The entire search, screening, and selection process is depicted in Fig. 1.

### Assessment of quality

The Joanna Briggs Institute (JBI) reviewers’ manual tools [53] were utilized to evaluate the quality of studies in this systematic review and meta-analysis. Quality scores ranged from 0 to 8, with scores of 7–8 denoting high quality, 4–6 suggesting moderate quality, and below 4 indicating low quality. A higher score reflects greater study quality.

### Data extraction

Table 1 outlines the characteristics of studies included in the meta-analysis, documenting author, publication year, country, article title, journal name, study design, and sample details (size, mean age, age range, percentage of female participants), along with measures of social media use and loneliness. Pearson’s bivariate correlation coefficient (*r*) was primarily used as the effect size; alternatively, other statistics (e.g., odds ratio, β coefficient, *t* and *F* values, chi-square value) were utilized following Lipsey & Wilson (2001) [54]. Effect sizes were extracted from independent samples, coding each correlation separately if multiple were reported within a single study. When multiple effect sizes were available for the same variables in a study, those from continuous, published, reliable, and valid scales were prioritized. If multiple outcomes were reported, the average correlation was computed using Fisher’s *z* and then back-transformed to *r* [55, 56].

**Table 1:** Characteristics of the included studies

	Author (year)	Study design	Nationality	Sample size (N)	Mean age (age range)	Female (%)	JBI	Social media use		Loneliness	Social media use and loneliness (*r*)
								Instrument	Type	Instrument	
1	An et al. (2020)	Cross-sectional	China	1804	NA (12–18)	56.3	8	Social network use questionnaire	Time	UCLA Loneliness Scale	-0.220
2	Aalbers et al. (2019)	Cross-sectional	Netherlands	132	20.4 (NA)	68.9	6	Self-Report Habit Index	Problem	Depression questionnaires (feelings of loneliness)	0.030
3	Al Omari et al. (2023)	Cross-sectional	Six Middle Eastern countries	1057	20 (15–24)	71.5	7	Open question: Time spent on social media	Time	UCLA Loneliness Scale	0.115
4	Akbari et al. (2023)	Cross-sectional	Iran	3375	15.5 (13–18)	67.1	8	Bergen social media addiction scale (BSMAS)	Problem	UCLA Loneliness Scale	0.340
5	Aydın et al. (2013)	Cross-sectional	Turkey	435	21.9 (NA)	63	5	Facebook usage information	Time	UCLA Loneliness Scale	-0.120
6	Azhari et al. (2022)	Cross-sectional	UK	41	17.8 (16–19)	100	6	1. Social media questionnaire 2. Social Media Disorder Scale (SMDS)	1. Time 2. Problem	Short Loneliness scale	1. 0.090 2. 0.450
7	Baltaci (2019)	Cross-sectional	Turkey	312	NA (19–25)	53	6	Social Media Addiction Scale (SMAS)	Problem	UCLA Loneliness Scale	0.185
8	Berryman et al. (2018)	Cross-sectional	USA	467	19.7 (NA)	71.1	8	Open question: Time spent on social media	Time	UCLA Loneliness Scale	0.055
9	Błachnio et al. (2016)	Cross-sectional	Poland	897	17.6 (NA)	66	8	Facebook Privacy Scale	Active	Jong-Gierveld Loneliness Scale	0.130
10	Charmaraman et al. (2022)	Cross-sectional	USA	586	12.5 (NA)	53	8	Problematic Internet Use scale (PRIUSS)	Problem	UCLA Loneliness Scale	0.295
11	Çiftci et al. (2023)	Cross-sectional	Turkey	1225	15.6 (14–18)	52	8	Bergen social media addiction scale (BSMAS)	Problem	UCLA Loneliness Scale	0.329
12	Dadiotis et al. (2021)	Cross-sectional	Greece	325	21.6 (NA)	81.8	4	1. Questions on time spent and number of SNSs accounts 2. Bergen Social Media Addiction Scale (BSMAS)	1. Time 2. Problem	UCLA Loneliness Scale	1.-0.002 2.-0.120
13	Dissing et al. (2022)	Cross-sectional	Denmark	815	21.6 (NA)	23	8	Tracked nighttime smartphone use	Time	UCLA Loneliness Scale	-0.155
14	Domoff et al. (2024)	Cross-sectional	USA	97	14.8 (13–18)	53.6	6	Social Media Disorder Scale (SMD)	Problem	UCLA Loneliness Scale	0.380
15	Durmuş et al. (2025)	Cross-sectional	Turkey	946	15.7 (14–18)	46	8	Social Media Disorder Scale (SMD)	Problem	UCLA Loneliness Scale	0.310
16	Eid et al. (2023)	Cross-sectional	Lebanon	379	16.1 (13–17)	64.9	8	Problematic Use of SNs Scale (PUS)	Problem	Jong-Gierveld Loneliness Scale	0.470
17	Ellis et al. (2020)	Cross-sectional	Canada	1054	16.7 (14–18)	76.4	7	Open question: Time spent on social media	Time	UCLA Loneliness Scale	0.040
18	Fernandes et al. (2021)	Cross-sectional	Multiple	1182	20.5 (16–25)	79.7	8	The Compulsive Internet Use Scale (CIUS)	Problem	UCLA Loneliness Scale	0.523
19	Frison & Eggermont (2020)	Cross-sectional	Belgium	1866	14.3 (12–19)	45	8	1. Daily time spent on Facebook 2. A multidimensional Facebook scales	1. Time 2. Active 3. Passive	Loneliness Scale	1.0.100 2.0.055 3.0.060
20	Gregory et al. (2023)	Cross-sectional	Australia	47	NA (16–24)	68.1	4	A multidimensional Facebook scales	1. Time 2. Active 3. Passive	Jong-Gierveld Loneliness Scale	1.0.060 2.0.138 3.0.198
21	Hood et al. (2018)	Cross-sectional	Australia	149	20.3 (17–25)	81.1	5	Open question: Time spent on social media	Time	The Social and Emotional Loneliness Scale	0.090
22	Karaköse et al. (2016)	Cross-sectional	Turkey	712	NA (14–18)	58.4	6	Bergen Facebook Addiction Scale (BFAS)	Problem	UCLA Loneliness Scale	0.130
23	Kilincel & Muratdagi (2021)	Cross-sectional	Turkey	1142	15.6 (12–18)	63.2	7	Social Media Disorder Scale (SMD)	Problem	UCLA Loneliness Scale	0.093
24	Kim et al. (2019)	Cross-sectional	USA	210	19.2 (NA)	71.3	8	Usage of the favorite celebrity’s social media	Time	UCLA Loneliness Scale	0.210
25	Kitiş et al. (2022)	Cross-sectional	Turkey	998	NA (16–24)	34.7	6	Social Media Addiction Scale (SMAS)	Problem	UCLA Loneliness Scale	-0.235
26	Koç & Ferneding (2007)	Cross-sectional	Turkey	758	NA (17–23)	55	6	Internet café usage	Time	UCLA Loneliness Scale	0.070
27	Kross et al. (2013)	Cross-sectional	USA	82	19.5 (NA)	64.6	8	Open question: How much have you used Facebook since the last time we asked?	Time	UCLA Loneliness Scale	0.220
28	Leung et al. (2011)	Cross-sectional	Hong Kong	374	16.5 (15–19)	58.3	8	Open question: Time spent on social media	Time	UCLA Loneliness Scale	0.736
29	Li et al. (2023)	Cross-sectional	China	106	20.5 (NA)	63.2	4	Facebook intensity scale	Time	UCLA Loneliness Scale	0.089
30	Lin et al. (2022)	Cross-sectional	China	390	19.4 (17–22)	64.6	8	The Active SNS Use Questionnaire	Active	The Emotional and Social Loneliness Scale (ESLS)	-0.210
31	Lin et al. (2023)	Cross-sectional	China	953	14.2 (12–17)	52.2	8	The Adolescent Problematic Mobile Social Media Usage Assessment Questionnaire	Problem	UCLA Loneliness Scale	0.290
32	Lyyra et al. (2022)	Cross-sectional	Finnish	3140	NA (11–15)	69	5	Problematic social media use (PSMU)	Problem	Health Behaviour in School-aged Children (HBSC)	0.190
33	MacDonald & Schermer (2021)	Cross-sectional	Canada	302	18.9 (18–25)	52.3	7	Open question: Time spent on social media	Time	UCLA Loneliness Scale	0.040
34	Magis-Weinberg et al. (2021)	Cross-sectional	Peru	735	13.3 (11–17)	63.2	6	Open question: Time spent on social media	Time	UCLA Loneliness Scale	0.220
35	Matook et al. (2015)	Cross-sectional	Australian	205	24.9 (NA)	61	6	Online Social Network Features	Passive	UCLA Loneliness Scale	0.420
36	Mousavi et al. (2023)	Cross-sectional	USA	215	15.6 (14–17)	47.9	8	Social media engagement	Time	UCLA Loneliness Scale	0.195
37	Nwosu et al. (2021)	Cross-sectional	Nigeria	600	19.7 (NA)	78.6	6	Internet addiction scale	Problem	UCLA Loneliness Scale	0.296
38	Obeid et al. (2023)	Cross-sectional	Lebanon	379	16.1 (15–18)	64.9	6	Problematic Use of SNs Scale (PUS)	Problem	Jong-Gierveld Loneliness Scale	0.470
39	Onat et al. (2025)	Cross-sectional	Turkey	90	15 (14–18)	38.9	8	Social Media Disorder Scale (SMD)	Problem	UCLA Loneliness Scale	0.429
40	Pouwels et al. (2021)	Cross-sectional	Netherlands	383	14 (NA)	54	8	Short-term social media use	Time	UCLA Loneliness Scale	-0.010
41	Rose et al. (2022)	Cross-sectional	USA	144	15.8 (12–18)	49	7	Open question: online interaction frequency	Time	UCLA Loneliness Scale	-0.260
42	Rutter et al. (2021)	Cross-sectional	USA	4592	14.6 (12–17)	47.9	8	Open question: Time spent on social media	Time	The Comprehensive Inventory of Thriving (CIT): Loneliness subscale	0.230
43	Sarman & Tuncay (2023)	Cross-sectional	Turkey	1176	15.6 (13–18)	58.4	6	Social media questionnaire	Time	UCLA Loneliness Scale	0.047
44	Scott et al. (2022)	Cross-sectional	Australian	329	20.1 (17–25)	68.1	6	Open question: Time spent on social media	Time	UCLA Loneliness Scale	0.080
45	Shawcroft et al. (2022)	Cross-sectional	USA	31,825	NA	46.9	8	Open question: Time spent on social media	Time	Monitoring the Future loneliness questionnaire	0.080
46	Stankovska et al. (2016)	Cross-sectional	North Macedonia	120	21.5 (21–23)	51	6	Scale for social network (SSN)	Problem	The Social and Emotional Loneliness Scale	0.401
47	Suzuki et al. (2021)	Cross-sectional	Japan	341	20 (NA)	70.7	6	Open question: Time spent on social media	Time	UCLA Loneliness Scale	-0.200
48	Teppers et al. (2014)	Cross-sectional	Belgium	256	15.9 (NA)	64	8	Open question: Time spent on Facebook	Time	Loneliness and Aloneness Scale for Children and Adolescents (LACA)	0.030
49	Tian et al. (2018)	Cross-sectional	China	5215	16.2 (10–23)	55.8	5	Facebook Usage scale	Time	UCLA Loneliness Scale	-0.070
50	Turhan Gürbüz et al. (2021)	Cross-sectional	Turkey	93	21 (15–24)	19.4	6	Social Media Addiction Scale (SMAS)	Problem	UCLA Loneliness Scale	0.391
51	Türk & Koçyiğit. (2025)	Cross-sectional	Turkey	342	12.7 (10–14)	50	6	Social Media Addiction Scale (SMAS)	Problem	UCLA Loneliness Scale	0.470
52	Wang et al. (2018)	Cross-sectional	Belgium	1188	14.3 (NA)	45	8	Multidimensional Scale of Facebook Use	1. Time 2. Active	The Loneliness Scale	1. 0.055 2. 0.045
53	Wang et al. (2022)	Cross-sectional	China	4172	16.4 (NA)	47.5	6	The adapted Facebook Intrusion Questionnaire	Problem	Three-Item Loneliness Scale	0.287
54	Xu et al. (2022)	Cross-sectional	China	830	14.5 (11–18)	54.6	8	Mobile Phone Addiction Type Scale (MPATS)	Problem	UCLA Loneliness Scale	0.339
55	Yang (2016)	Cross-sectional	USA	208	19.4 (18–25)	78	8	Open question: Instagram use for times a week	Time	UCLA Loneliness Scale	-0.080
56	Yang et al. (2020)	Cross-sectional	USA	219	18.3 (NA)	74	8	Compulsive Internet Use Scale (CIUS)	Time	The Three-Item Loneliness Scale	0.020
57	Ye & Lin (2015)	Cross-sectional	China	260	20.1 (18–24)	67.7	6	Preference for online social interaction (POSI)	Time	UCLA Loneliness Scale	0.200
58	Yildiz & Seferoğlu (2019)	Cross-sectional	Turkey	580	22.9 (NA)	59.8	8	1. Social Network Sites Usage Scale 2. Social Media Disorder Scale (SMDS)	1. Time 2. Problem	UCLA Loneliness Scale	1. 0.053 2. 0.309
59	Yurdagül et al. (2021)	Cross-sectional	Turkey	491	15.9 (14–119)	58.7	4	Bergen Facebook Addiction Scale (BFAS)	Problem	UCLA Loneliness Scale	0.140
NA, not applicable											

### Statistical analysis

To explore the relationship between social media use and loneliness, we employed the Pearson correlation coefficient (*r*). Social media usage was analyzed as a continuum and categorized into problematic use, time spent, active use, and passive use based on questionnaire scores. Data analysis was executed using Comprehensive Meta-Analysis Version 3.3, utilizing a random effects model to accommodate heterogeneity across studies. We calculated the variance of* r* to derive the standard error for each correlation, ensuring each effect size was accompanied by a 95% confidence interval.

We assessed heterogeneity in effect sizes using the *Q* statistic and* I*^*2*^ index, with *I*^*2*^ values of 25%, 50%, and 75% indicating low, moderate, and high heterogeneity, respectively. Depending on the level of heterogeneity, we applied either a fixed-effects model (*I*^*2*^ < 50%) or a random-effects model (*I*^*2*^ ≥ 50%) to compute pooled correlations and 95% confidence intervals [57]. To identify potential sources of heterogeneity, we examined both categorical moderators (e.g., types of social media use: problematic use, time spent, active use, and passive use) and continuous moderators (e.g., geographic region, the percentage of female participants, and participants’ mean age). Subgroup analyses were used for categorical variables, while meta-regression was conducted for continuous variables. All moderator analyses were conducted with the aim of evaluating their influence on the association between social media use and loneliness.

To evaluate potential publication bias in studies examining the relationship between social media use and loneliness, we applied both the Fail-safe N analysis [58] and Egger’s linear regression method [59]. The Fail-safe N determines how many additional non-significant studies would be needed to render a significant meta-analytic finding insignificant, with robustness confirmed if this number surpasses the 5 k + 10 threshold [60]. Egger’s method analyzes the symmetry of the funnel plot; a significant asymmetry indicates potential small-study bias. We also conducted sensitivity analyses to ensure the robustness of our findings.

### Ethical considerations

We have detailed our search and data extraction processes, adhering to the Journal Article Reporting Standards and meta-analysis reporting standards as outlined by Appelbaum et al. (2018) [61]. All data, analysis code, and research materials used in this study are available upon request. This study is registered with PROSPERO (CRD42024572948) and received ethical approval from the Institutional Review Board at National Taiwan Normal University (ID: NTNUREC-202408HS010).

## Results

### Results of the literature search

Figure 1 presents a flowchart of the study selection process, which yielded 59 empirical, quantitative, observational studies comprising a total of 81,344 participants, 59.5% of whom were female. These studies contributed 67 independent effect sizes. The mean age of participants was 17.6 years (SD = 2.9), with reported ages ranging from 12.5 to 24.9 years. The samples were geographically diverse, including 28 studies conducted in Western countries and 31 in Eastern or multi-country contexts. All included studies employed self-report measures to examine the association between social media use and loneliness.

The Bergen Social Media Addiction Scale (BSMAS) was the predominant tool for assessing problematic social media use, followed by the Problematic Internet Use Scale (PIUSS) and Compulsive Internet Use Scale (CIUS). Daily social media usage was commonly measured by self-reported hours, with the Facebook Usage Scale being the next most utilized method. Assessments of passive or active internet use often employed Multidimensional Facebook Scales, the Active SNS Use Questionnaire, and Online Social Network Features. For loneliness, the University of California Los Angeles (UCLA) loneliness scale was most frequently used, with the Jong-Gierveld Loneliness Scale as a secondary measure. Details on each study are compiled in Table 1.

### Associations between social media use and loneliness

Meta-analytic results using a random-effects model revealed a significant association between social media use and loneliness, yielding a pooled correlation coefficient of *r* = 0.16 (95% CI [0.12–0.20], *p* < 0.001). Nevertheless, considerable heterogeneity was detected across studies (*I*^*2*^ = 97.01%), indicating substantial variability in effect sizes (Table 2). In light of this high heterogeneity, we conducted subgroup and meta-regression analyses to identify potential moderators. These included specific types of social media use (problematic use, time spent, active use, and passive use), as well as geographic region, the percentage of female participants, and participants’ mean age.

**Table 2:** Summary of meta-analytic results on associations between social media use and loneliness

Mental health variable	Social media use type	Number of studies	Number of participants	*r*	*I*^*2*^* (%)*	95% CI	*p-value*
Loneliness	Overall	67	81,344	0.16	97.01	0.12 – 0.20	< 0.001
	Problematic	26	25,719	0.29	95.69	0.22 – 0.35	< 0.001
	Time use	33	57,458	0.07	96.07	0.01 – 0.12	0.014
	Active	5	4,388	0.02	87.86	-0.08 – 0.12	0.661
	Passive	3	2,118	0.23	92.82	-0.06 – 0.48	0.116

Participant numbers reported in the subcategories may overlap because several studies included more than one type of social media use. As a result, the combined subcategory totals may be greater than the overall participant count

### Subgroup analysis between social media use and loneliness

To further explore the sources of heterogeneity, social media use was categorized into four distinct types: problematic use, time spent, active use, and passive use. Subgroup analyses revealed a significant positive association between problematic social media use and loneliness (*r* = 0.29, 95% CI [0.22–0.35], *p* < 0.001), as well as between time spent on social media and loneliness (*r* = 0.07, 95% CI [0.01–0.12], *p* = 0.01). In contrast, neither active use (*r* = 0.02, 95% CI [–0.08–0.12], *p* = 0.66) nor passive use (*r* = 0.23, 95% CI [–0.06–0.48], *p* = 0.12) was significantly associated with loneliness. The test for subgroup differences was significant (*Q*_between_ = 34.51; *p* < 0.001), indicating that the type of social media use moderated the strength of the association with loneliness (see Table 2).

### Results of the meta-regression

**Fig. 2:**
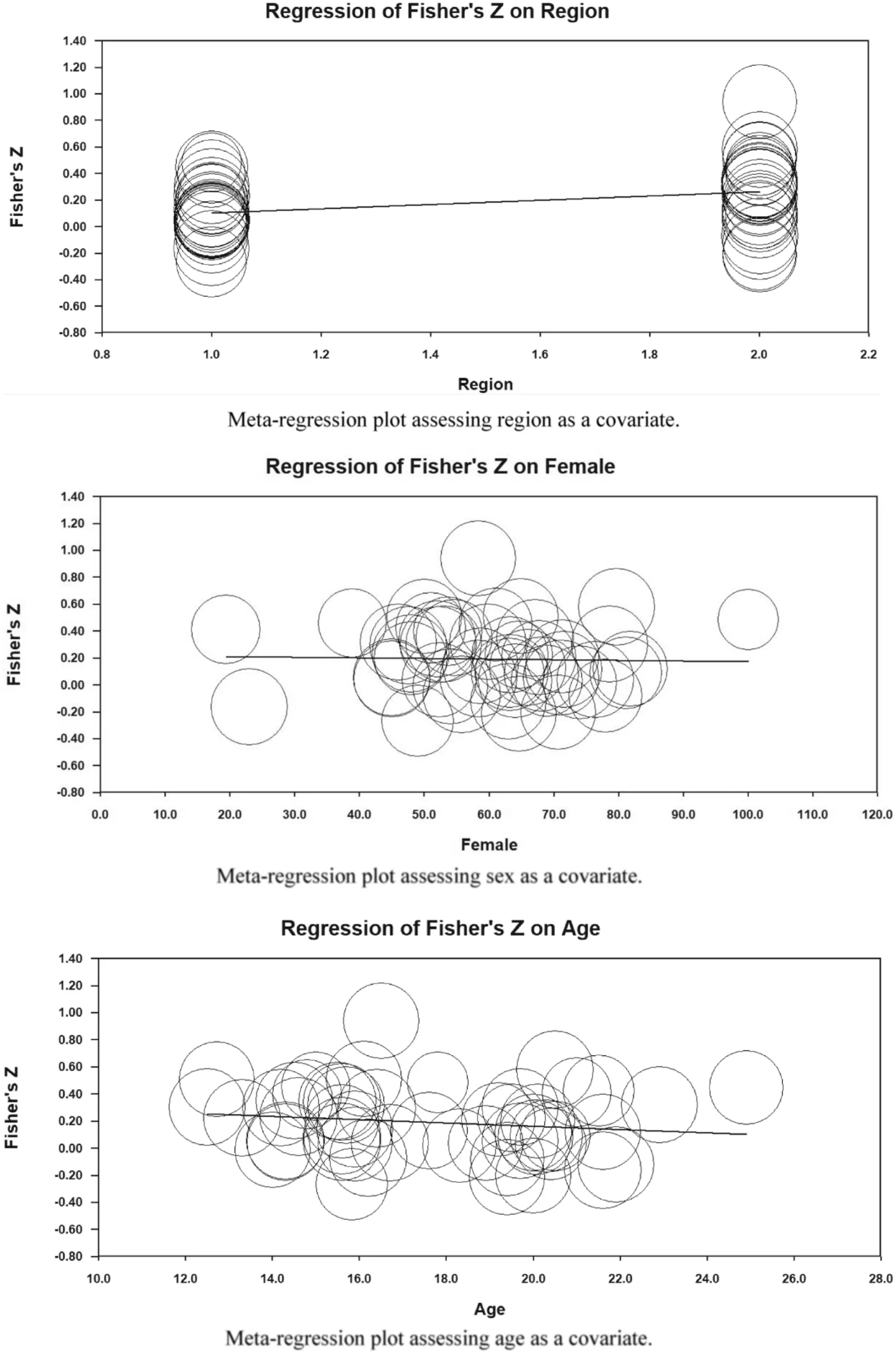
Meta-regression plot assessing covariates

In addition to the subgroup analyses, meta-regression was employed to assess whether geographic region, the percentage of female participants, and participants’ mean age moderated the association between social media use and loneliness. The analysis revealed no significant moderating effects (*p*-values: 0.11, 0.87, and 0.51, respectively), indicating that these variables did not explain the observed between-study heterogeneity. Meta-regression plots illustrating these findings are presented in Fig. 2.

### Publication bias of social media use and loneliness

**Fig. 3:**
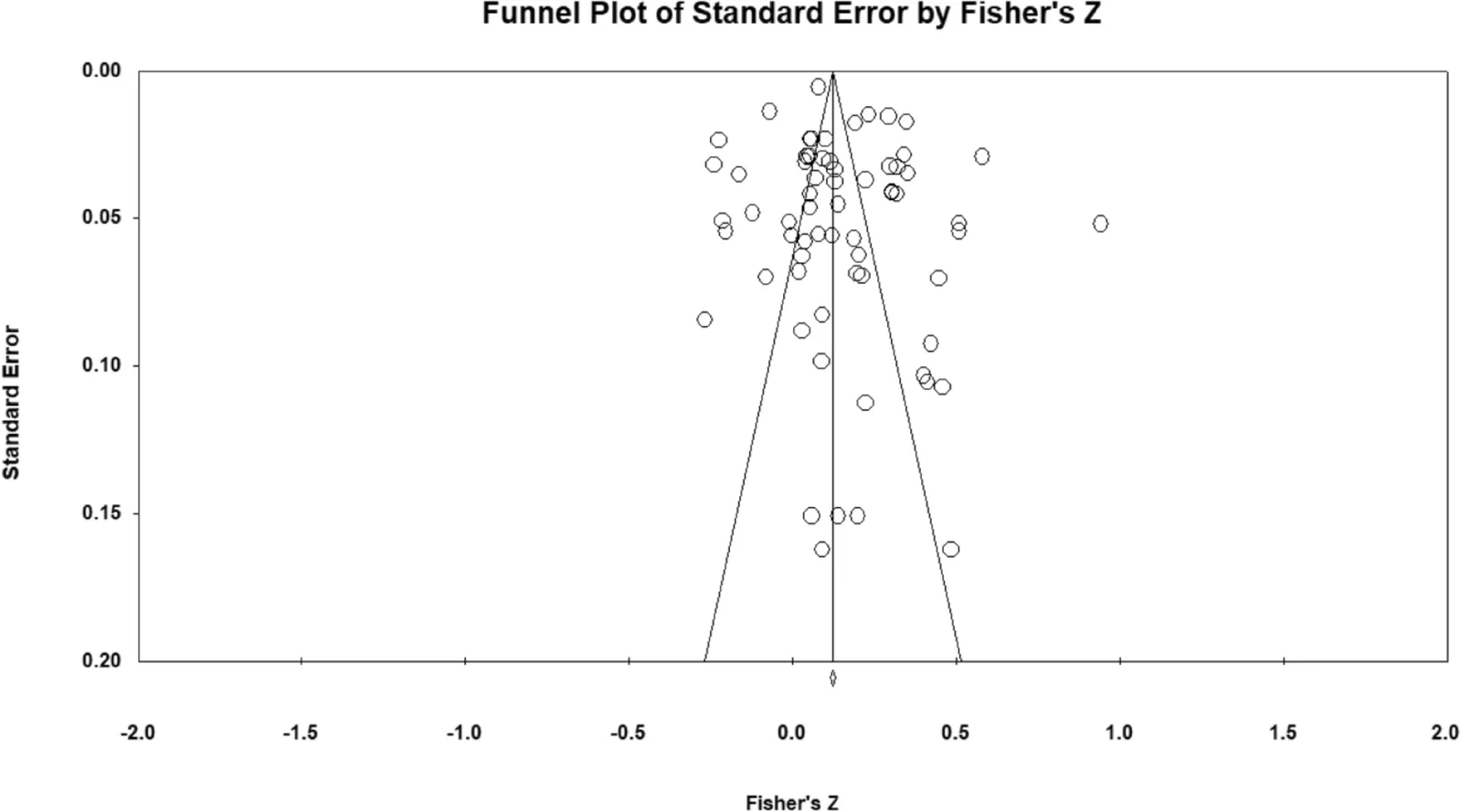
Funnel plot of social media use and loneliness

The publication bias analysis for the association between social media use and loneliness, conducted using the Fail-safe N method, indicated that over 4,728 null-result studies would be required to reduce the significant effect size estimates to non-significance. Additionally, Egger’s regression test indicated no statistically significant asymmetry (*p* = 0.27) in the funnel plot distribution (Fig. 3).

### Sensitivity analysis

To evaluate the robustness of our findings, we conducted multiple sensitivity analyses in line with established best practices. First, we performed leave-one-out analyses, sequentially excluding each study to assess its influence on the pooled effect size; the consistency of results indicated that no single study exerted undue influence on the overall outcome. Second, we excluded studies rated as having low methodological quality (JBI ≤ 4) and repeated the meta-analysis. The pooled effect sizes remained stable, underscoring resilience across study quality. Collectively, these analyses support the stability and robustness of our primary conclusions.

## Discussion

To our knowledge, this study represents one of the first comprehensive attempts to clarify how different patterns of social media use (problematic, time spent, active, and passive) are associated with adolescent loneliness. Overall, the meta-analysis revealed a small yet statistically significant association, consistent with prior work highlighting the nuanced interplay between social media and psychosocial outcomes [62, 63]. Given that pooled estimates are correlational, these findings should be interpreted as evidence of association rather than causation. While the modest effect size indicates that social media use shows only a limited association with loneliness, the findings suggest that specific usage patterns, rather than overall exposure, may be differentially associated with adolescents’ well-being. Heterogeneity across studies, likely due to varying conceptualizations and measurement strategies, also highlights the need for more consistent approaches to studying this phenomenon [64].

Among the four usage patterns, problematic social media use showed the strongest association with loneliness. This finding supports previous work indicating that maladaptive, compulsive, or dependency-driven engagement is more strongly linked to negative psychosocial outcomes than mere usage duration [65–67]. Two processes may help account for this stronger association. First, excessive reliance on social media for gratification may be associated with the displacement of face-to-face connections, weakening real-world support networks and heightening feelings of isolation [63, 68]. Second, the association may be bidirectional: loneliness may drive individuals toward maladaptive online behaviors, which in turn may be associated with heightened loneliness, creating a reinforcing cycle [35, 43]. Importantly, loneliness may also function as a key motivational factor underlying intensified or problematic engagement with social media. Adolescents experiencing elevated loneliness may turn to online platforms as a compensatory strategy to fulfill unmet social needs or seek perceived social connection, consistent with the stimulation and social compensation hypotheses [34, 35]. Longitudinal evidence further suggests that baseline loneliness is prospectively associated with subsequent increases in problematic social media use and declines in well-being [33, 67]. Such findings support the plausibility of reverse or reciprocal pathways between loneliness and maladaptive engagement [66]. Therefore, the pooled correlation observed in the present study is best interpreted as reflecting a dynamic and potentially bidirectional association rather than a unidirectional causal process. This reciprocal process emphasizes the importance of considering both vulnerability and consequence in understanding problematic use.

By contrast, the amount of time spent on social media demonstrated only negligible associations with loneliness. This suggests that the quality of engagement may be more closely related to loneliness than its quantity [69]. Adolescents may spend substantial time online without necessarily experiencing negative effects, provided that interactions remain positive and socially supportive. Additionally, methodological variability in how “time spent” is assessed, whether through self-report or retrospective estimates, may further dilute observed effects [70, 71]. These findings reinforce the growing consensus that intervention efforts should be cautious in relying solely on “screen time” as an indicator of psychosocial risk, given the modest magnitude of association observed.

Similarly, both active and passive forms of social media use showed weak or non-significant associations with loneliness. While active use has been theorized to enhance connectedness and passive use to foster unfavorable social comparisons [72, 73], our results suggest that these distinctions may not adequately capture adolescents’ experiences. Several factors may contribute to this. First, individual differences such as personality traits and pre-existing social needs may moderate how each form of use is associated with well-being [74]. Second, most adolescents engage in blended patterns of active and passive behaviors, making it difficult to isolate their unique effects. Third, the quality and context of online interactions may overshadow the simple dichotomy between active and passive use [73, 74]. Taken together, these findings highlight that social media’s association with loneliness depends less on activity type per se than on the relational value embedded within interactions.

Our subgroup analyses further strengthen these conclusions. Consistent with prior meta-analyses, problematic use consistently displayed the strongest association with loneliness, regardless of study characteristics [19, 50]. This convergence across independent syntheses underscores the robustness of the problematic use–loneliness link and indicates that maladaptive engagement is more strongly correlated with loneliness than other usage patterns, although the directionality of this relationship cannot be determined from the available data.

Several limitations should be acknowledged. First, substantial heterogeneity across studies may reflect inconsistencies in how social media use and loneliness are conceptualized and measured [19, 64]. Future work would benefit from standardized and multidimensional assessments that distinguish between usage patterns, motivations, and contexts. Second, the reliance on self-report data introduces risks of recall bias and social desirability effects. Incorporating objective digital trace data, such as usage logs or passive sensing, would enhance validity. Third, the cross-sectional design of most included studies limits causal inference. Accordingly, the present findings should be interpreted as evidence of association rather than causation. Longitudinal and experimental research is necessary to clarify temporal ordering and test for potential bidirectionality [48].

In conclusion, this meta-analysis indicates that while social media use overall shows only a modest association with adolescent loneliness, problematic use consistently emerges as the pattern most strongly associated with loneliness. However, it remains unclear whether problematic use contributes to increased loneliness, whether loneliness predicts intensified engagement, or whether both processes operate simultaneously. These findings highlight the importance of further longitudinal and experimental research before firm intervention priorities can be established. For researchers, the results underscore the need to move beyond simplistic usage metrics to examine the quality, context, and function of online interactions. For practitioners and policymakers, the findings suggest that prevention efforts may benefit from addressing both adolescents’ underlying social vulnerabilities and their patterns of digital engagement, while acknowledging that the temporal and causal nature of these associations remains to be clarified.

## Data Availability

No datasets were generated or analysed during the current study.

## References

[1] Kaplan AM, Haenlein M (2010) Users of the world, unite! The challenges and opportunities of social media. Bus Horiz 53(1):59–68

[2] Chassiakos YR, Stager M (2020) Chapter 2 - Current trends in digital media: how and why teens use technology. In: Moreno MA, Hoopes AJ (eds) Technology and adolescent health. Academic Press, pp 25–56

[3] Smith D, Leonis T, Anandavalli S (2021) Belonging and loneliness in cyberspace: impacts of social media on adolescents’ well-being. Aust J Psychol 73(1):12–23

[4] Vogels EA, Gelles-Watnick R, Massarat N (2022) Teens, social media and technology 2022

[5] Perlman D, Peplau LA (1981) Toward a social psychology of loneliness. Pers Relat 3:31–56

[6] Thomas L, Orme E, Kerrigan F (2020) Student loneliness: the role of social media through life transitions. Comput Educ 146:103754

[7] Ellard OB, Dennison C, Tuomainen H (2023) Interventions addressing loneliness amongst university students: a systematic review. Child Adolesc Mental Health 28(4):512–523 10.1111/camh.12614 36496554

[8] Neto R, Golz N, Polega M (2015) Social media use, loneliness, and academic achievement: a correlational study with urban high school students. J Res Educ 25(2):28–37

[9] Urke HB, Larsen TB, Kristensen SME (2023) Preventing loneliness and reducing dropout: results from the complete intervention study in upper secondary schools in Norway. Int J Environ Res Public Health 20(13):6299 37444146 10.3390/ijerph20136299 PMC10341405

[10] Danneel S et al (2020) Loneliness, social anxiety symptoms, and depressive symptoms in adolescence: longitudinal distinctiveness and correlated change. J Youth Adolesc 49:2246–2264 32918664 10.1007/s10964-020-01315-w

[11] Wang J et al (2025) Loneliness, internalizing and externalizing problems, and suicidal ideation among Chinese adolescents: a longitudinal mediation analysis. J Adolesc Health 76(1):96–104 39365230 10.1016/j.jadohealth.2024.08.010

[12] An R, Jiang Y, Bai X (2020) The relationship between adolescent social network use and loneliness: multiple mediators of online positive feedback and positive emotions. Chin J Clin Psychol 28(4):824–833

[13] Kim Y, Sohn D, Choi SM (2011) Cultural difference in motivations for using social network sites: a comparative study of American and Korean college students. Comput Hum Behav 27(1):365–372

[14] Laursen B, Hartl AC (2013) Understanding loneliness during adolescence: developmental changes that increase the risk of perceived social isolation. J Adolesc 36(6):1261–1268 23866959 10.1016/j.adolescence.2013.06.003

[15] Kim Y, Lee M (2025) Social media use and loneliness among adolescents: the moderating role of media literacy. Online Inf Rev 49(3):585–599

[16] Lisitsa E et al (2020) Loneliness among young adults during COVID-19 pandemic: the mediational roles of social media use and social support seeking. J Soc Clin Psychol 39(8):708–726

[17] Radovic A et al (2017) Depressed adolescents’ positive and negative use of social media. J Adolesc 55:5–15 27997851 10.1016/j.adolescence.2016.12.002 PMC5485251

[18] Hancock J, et al (2022) Psychological well-being and social media use: a meta-analysis of associations between social media use and depression, anxiety, loneliness, eudaimonic, hedonic and social well-being. Anx Lonel Eudaim Hedon Soc Well-Being (March 9 , 2022)

[19] Huang C (2017) Time spent on social network sites and psychological well-being: a meta-analysis. Cyberpsychol Behav Soc Netw 20(6):346–354 28622031 10.1089/cyber.2016.0758

[20] Riehm KE et al (2019) Associations between time spent using social media and internalizing and externalizing problems among US youth. JAMA Psychiat 76(12):1266–1273 10.1001/jamapsychiatry.2019.2325 PMC6739732 31509167

[21] Berryman C, Ferguson CJ, Negy C (2018) Social media use and mental health among young adults. Psychiatr Q 89(2):307–314 29090428 10.1007/s11126-017-9535-6

[22] Frison E, Eggermont S (2020) Toward an integrated and differential approach to the relationships between loneliness, different types of Facebook use, and adolescents’ depressed mood. Commun Res 47(5):701–728

[23] Gregory L et al (2023) Does social media usage ameliorate loneliness in rural youth? A cross sectional pilot study. BMC Psychiatry 23(1):371 37237363 10.1186/s12888-023-04849-y PMC10214363

[24] Aydın GS, Muyan M, Demir A (2013) The investigation of Facebook usage purposes and shyness, loneliness. Procedia-Soc Behav Sci 93:737–741

[25] Dadiotis A et al (2021) Validation of the Greek version of the Bergen social media addiction scale in undergraduate students. EMBnet J. 10.14806/ej.26.1.975 34722220 PMC8553118

[26] Tian Y et al (2018) Association between specific internet activities and life satisfaction: the mediating effects of loneliness and depression. Front Psychol 9:1181 30050484 10.3389/fpsyg.2018.01181 PMC6050461

[27] Shannon H et al (2022) Problematic social media use in adolescents and young adults: systematic review and meta-analysis. JMIR Ment Health 9(4):e33450 35436240 10.2196/33450 PMC9052033

[28] Bányai F et al (2017) Problematic social media use: results from a large-scale nationally representative adolescent sample. PLoS ONE 12(1):e0169839 28068404 10.1371/journal.pone.0169839 PMC5222338

[29] Baltaci Ö (2019) The predictive relationships between the social media addiction and social anxiety, loneliness, and happiness. Int J Prog Educ 15(4):73–82

[30] Charmaraman L et al (2022) Examining early adolescent positive and negative social technology behaviors and well-being during the COVID-19 pandemic. Technol Mind Behavior. 10.1037/tmb0000062 PMC9769924 36561093

[31] Lin S et al (2024) Family matters more than friends on problematic social media use among adolescents: mediating roles of resilience and loneliness. Int J Ment Health Addict 22(5):2907–2925 10.1007/s11469-023-01026-w PMC9933806 36811077

[32] Chen Y et al (2022) Problematic social media use and depressive outcomes among college students in China: observational and experimental findings. Int J Environ Res Public Health 19(9):4937 35564330 10.3390/ijerph19094937 PMC9099455

[33] Marttila E, Koivula A, Räsänen P (2021) Does excessive social media use decrease subjective well-being? A longitudinal analysis of the relationship between problematic use, loneliness and life satisfaction. Telemat Inform 59:101556

[34] Valkenburg PM, Peter J (2007) Online communication and adolescent well-being: testing the stimulation versus the displacement hypothesis. J Comput-Mediat Commun 12(4):1169–1182

[35] Nowland R, Necka EA, Cacioppo JT (2018) Loneliness and social internet use: pathways to reconnection in a digital world? Perspect Psychol Sci 13(1):70–87 28937910 10.1177/1745691617713052

[36] Verduyn P et al (2017) Do social network sites enhance or undermine subjective well-being? a critical review. Soc Issues Policy Rev 11(1):274–302

[37] Lin S et al (2022) Active social network sites use and loneliness: the mediating role of social support and self-esteem. Curr Psychol 41(3):1279–1286

[38] Hanley SM, Watt SE, Coventry W (2019) Taking a break: the effect of taking a vacation from Facebook and Instagram on subjective well-being. PLoS ONE 14(6):e0217743 31170206 10.1371/journal.pone.0217743 PMC6553853

[39] Matook S, Cummings J, Bala H (2015) Are you feeling lonely? The impact of relationship characteristics and online social network features on loneliness. J Manag Inf Syst 31(4):278–310

[40] Frison E, Eggermont S (2016) Exploring the relationships between different types of Facebook use, perceived online social support, and adolescents’ depressed mood. Soc Sci Comput Rev 34(2):153–171

[41] Primack BA et al (2017) Social media use and perceived social isolation among young adults in the US. Am J Prev Med 53(1):1–8 28279545 10.1016/j.amepre.2017.01.010 PMC5722463

[42] Fish JN et al (2020) “I’m kinda stuck at home with unsupportive parents right now”: LGBTQ youths’ experiences with COVID-19 and the importance of online support. J Adolesc Health 67(3):450–452 32591304 10.1016/j.jadohealth.2020.06.002 PMC7309741

[43] Song H et al (2014) Does Facebook make you lonely?: a meta analysis. Comput Hum Behav 36:446–452

[44] Liu D, Baumeister RF (2016) Social networking online and personality of self-worth: a meta-analysis. J Res Pers 64:79–89

[45] Lopez-Fernandez O et al (2023) Problematic internet use among adults: a cross-cultural study in 15 countries. J Clin Med 12(3):1027 36769675 10.3390/jcm12031027 PMC9917388

[46] Luo T et al (2021) Determination the cut-off point for the Bergen social media addiction (BSMAS): diagnostic contribution of the six criteria of the components model of addiction for social media disorder. J Behav Addict 10(2):281–290 34010148 10.1556/2006.2021.00025 PMC8996805

[47] Zhou W et al (2023) Problematic social media use and mental health risks among first-year Chinese undergraduates: a three-wave longitudinal study. Front Psychiatry 14:1237924 37743982 10.3389/fpsyt.2023.1237924 PMC10512716

[48] Coyne SM et al (2020) Does time spent using social media impact mental health?: an eight year longitudinal study. Comput Hum Behav 104:106160

[49] Vahedi Z, Zannella L (2021) The association between self-reported depressive symptoms and the use of social networking sites (SNS): a meta-analysis. Curr Psychol 40(5):2174–2189

[50] Liu M et al (2022) Time spent on social media and risk of depression in adolescents: a dose–response meta-analysis. Int J Environ Res Public Health. 10.3390/ijerph19095164 35564559 PMC9103874

[51] Saadati HM et al (2021) Association between internet addiction and loneliness across the world: a meta-analysis and systematic review. SSM Popul Health 16:100948 34754896 10.1016/j.ssmph.2021.100948 PMC8563346

[52] Zhang L et al (2022) Social networking site use and loneliness: a meta-analysis. J Psychol 156(7):492–511 35981234 10.1080/00223980.2022.2101420

[53] Peters MDJ et al (2015) Guidance for conducting systematic scoping reviews. JBI Evid Implement 13(3):141–146 10.1097/XEB.0000000000000050 26134548

[54] Lipsey MW, Wilson DB (2001) Practical meta-analysis. SAGE publications, Inc.

[55] Borenstein M et al (2009) Effect sizes for continuous data. The Handb Res Synth Meta-Anal 2:221–235

[56] Lien YJ et al (2023) The power of knowledge: how mental health literacy can overcome barriers to seeking help. Am J Orthopsychiatry. 10.1037/ort0000708 37917500

[57] Higgins JP, Thompson SG (2002) Quantifying heterogeneity in a meta-analysis. Stat Med 21(11):1539–1558 12111919 10.1002/sim.1186

[58] Orwin RG (1983) A fail-safe N for effect size in meta-analysis. J Educ Stat 8(2):157–159

[59] Sterne JA, Egger M, Smith GD (2001) Investigating and dealing with publication and other biases in meta-analysis. BMJ 323(7304):101–105 11451790 10.1136/bmj.323.7304.101 PMC1120714

[60] Rosenberg MS (2005) The file-drawer problem revisited: a general weighted method for calculating fail-safe numbers in meta-analysis. Evolution 59(2):464–468 15807430

[61] Appelbaum M et al (2018) Journal article reporting standards for quantitative research in psychology: the APA publications and communications board task force report. Am Psychol 73(1):3 29345484 10.1037/amp0000191

[62] Uhls YT, Ellison NB, Subrahmanyam K (2017) Benefits and costs of social media in adolescence. Pediatrics 140(Supplement_2):S67–S70 29093035 10.1542/peds.2016-1758E

[63] Twenge JM, Campbell WK (2018) Associations between screen time and lower psychological well-being among children and adolescents: evidence from a population-based study. Prev Med Rep 12:271–283 30406005 10.1016/j.pmedr.2018.10.003 PMC6214874

[64] Appel M, Marker C, Gnambs T (2020) Are social media ruining our lives? A review of meta-analytic evidence. Rev Gen Psychol 24(1):60–74

[65] Hussain Z, Griffiths MD (2018) Problematic social networking site use and comorbid psychiatric disorders: a systematic review of recent large-scale studies. Front Psychiatry 9:686 30618866 10.3389/fpsyt.2018.00686 PMC6302102

[66] Moretta T, Buodo G (2020) Problematic Internet use and loneliness: how complex is the relationship? A short literature review. Curr Addict Rep 7:125–136

[67] Marino C et al (2018) The associations between problematic Facebook use, psychological distress and well-being among adolescents and young adults: a systematic review and meta-analysis. J Affect Disord 226:274–281 29024900 10.1016/j.jad.2017.10.007

[68] Keles B, McCrae N, Grealish A (2020) A systematic review: the influence of social media on depression, anxiety and psychological distress in adolescents. Int J Adolesc Youth 25(1):79–93

[69] O’Day EB, Heimberg RG (2021) Social media use, social anxiety, and loneliness: a systematic review. Comput Hum Behav Rep 3:100070

[70] Griffioen N et al (2021) Everyone does it—differently: a window into emerging adults’ smartphone use. Humanit Soc Sci Commun 8(1):1–1138617731

[71] Twenge JM, Martin GN (2020) Gender differences in associations between digital media use and psychological well-being: evidence from three large datasets. J Adolesc 79:91–102 31926450 10.1016/j.adolescence.2019.12.018

[72] Verduyn P et al (2015) Passive Facebook usage undermines affective well-being: experimental and longitudinal evidence. J Exp Psychol Gen 144(2):480 25706656 10.1037/xge0000057

[73] Thorisdottir IE et al (2019) Active and passive social media use and symptoms of anxiety and depressed mood among Icelandic adolescents. Cyberpsychol Behav Soc Netw 22(8):535–542 31361508 10.1089/cyber.2019.0079

[74] Clark JL, Algoe SB, Green MC (2018) Social network sites and well-being: the role of social connection. Curr Dir Psychol Sci 27(1):32–37

